# Supplementary material for: Strong angular and spectral narrowing of electroluminescence in an integrated Tamm-plasmon-driven halide perovskite LED
Source: Nat Commun. 2024 Jul 10;15:5802. doi: 10.1038/s41467-024-49838-1 (PMC11237071; doi:10.1038/s41467-024-49838-1)
Supplement: Supplementary file 1 — Supplementary Information [file 41467_2024_49838_MOESM1_ESM.pdf]

Supplementary Information

Strong angular and spectral narrowing of electroluminescence in an integrated Tamm-plasmon-driven halide perovskite LED

Zher Ying Ooi [1], Alberto Jiménez-Solano [2,3], Krzysztof Gałkowski [4,5,6], Yuqi Sun [4], Jordi Ferrer Orri [4,7], Kyle Frohna [4], Hayden Salway [1], Simon Kahmann [1,4], Shenyu Nie [1], Guadalupe Vega [3,8], Shaoni Kar [4], Michał P. Nowak [9], Sebastian Maćkowski [5], Piotr Nyga [9], Caterina Ducati [7], Neil C. Greenham [4], Bettina V. Lotsch [2,10,11], Miguel Anaya [1,8]*, Samuel D. Stranks [1,4]*

1. Department of Chemical Engineering and Biotechnology, University of Cambridge, Cambridge, UK
2. Max Planck Institute for Solid State Research, Heisenbergstrasse 1, 70569 Stuttgart, Germany
3. Departamento de Física, Universidad de Córdoba, Edificio Einstein (C2), Campus de Rabanales, 14071 Córdoba, Spain
4. Cavendish Laboratory, University of Cambridge, Cambridge, UK
5. Institute of Physics, Faculty of Physics, Astronomy and Informatics, Nicolaus Copernicus University, Toruń, Poland
6. Department of Experimental Physics, Faculty of Fundamental Problems of Technology, Wroclaw University of Science and Technology, Wroclaw, Poland
7. Department of Materials Science and Metallurgy, University of Cambridge, Cambridge, UK
8. Departamento Física de la Materia Condensada, Instituto de Ciencia de Materiales de Sevilla, Universidad de Sevilla−CSIC, Calle Américo Vespucio 49, Sevilla 41012, Spain.
9. Institute of Optoelectronics, Military University of Technology, Warsaw, Poland
10. Department of Chemistry, Ludwig-Maximilians-Universität (LMU), Butenandtstrasse 5-13, 81377 Munich, Germany
11. e-conversion, Lichtenbergstrasse 4a, 85748 Garching, Germany

*sds65@cam.ac.uk, *ma811@cam.ac.uk

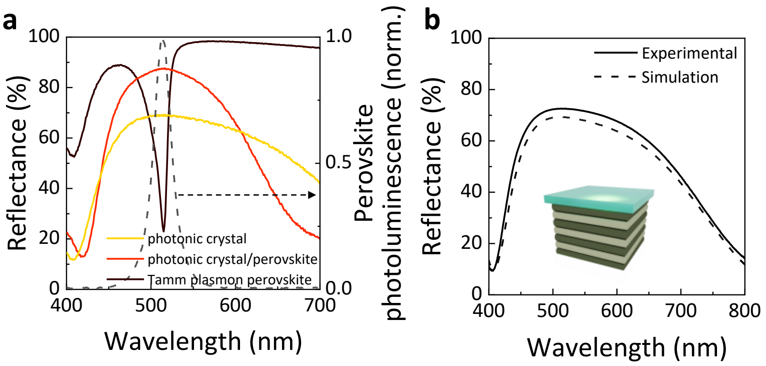

Supplementary Figure 1 | Modelling and optimization of perovskite-based Tamm plasmon structure. a, Reflectance of 1-dimensional photonic crystal, photonic crystal/perovskite stack and Tamm-plasmon-perovskite structure simulated with transfer matrix model. Perovskite film photoluminescence measured with a fluorimeter. **b,** Comparison of simulated and experimentally fabricated optical stopband of photonic crystal.

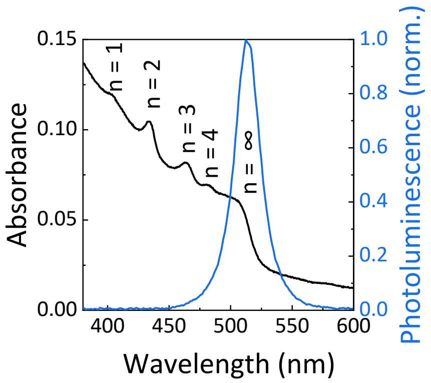

Supplementary Figure 2 | Absorbance and photoluminescence spectra of quasi-2D perovskite film without the 18-crown-6 additive. Quasi-2D perovskite is a general category of any perovskite with a mixed number of inorganic octahedral layers, n up to n > 5 layers sandwiched between the organic cation ligands [1]. The carriers funnel from smaller n (higher bandgap) to larger n (lower bandgap), accumulate at the largest n recombination centres and show only single narrow photoluminescence peak at the largest n recombination centres and show only single narrow photoluminescence peak at the large-n bandgap.

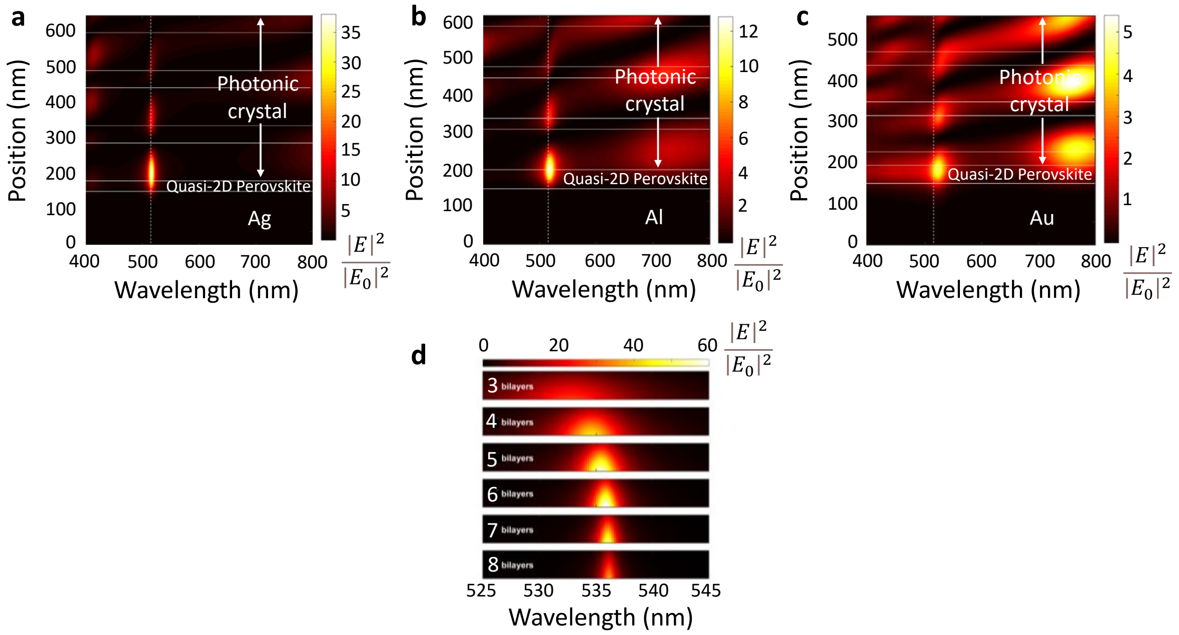

Supplementary Figure 3 | Simulation comparison between different metals and number of photonic crystal layer pairs. Optimization for perovskite-based Tamm plasmon structure is repeated for noble metals to find the highest possible electric field enhancements. The Tamm-plasmon-perovskite structure with highest electric field enhancement is compared between **(a)** Ag, **(b)** Al and **(c)** Au as the plasmonic metal. The quasi-2D perovskite, photonic crystal materials and number of photonic crystal layer pairs were kept constant at 3 bilayers in this comparison. **d,** Spectral and spatial distribution of electric field intensity within the perovskite layer, as a function of the number of bilayers hosting the perovskite layer. Above 6 bilayers, due to spatial and spectral narrowing of the electric field intensity, more careful optimisation is required to confine the maximum electric field within the perovskite layer.

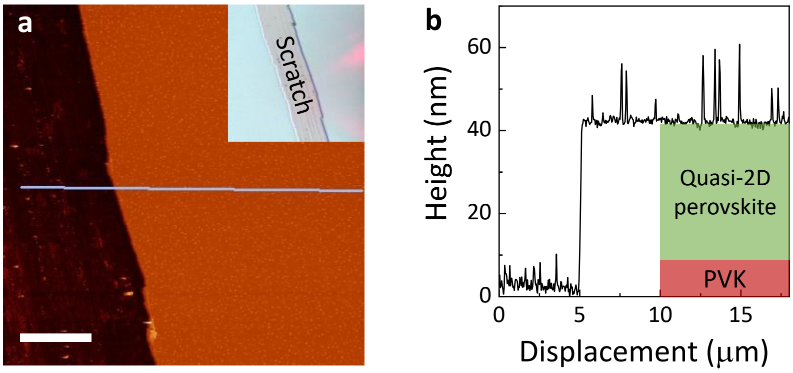

Supplementary Figure 4 | Thin film thickness measurement. (a-b) Thickness of quasi-2D perovskite and polyvinylcarbazole (PVK) on photonic crystals substrate measured under atomic force microscope (AFM) by scanning through a razor blade scratch to remove both layers. The scale bar shows 4 μm. Inset: optical image of scratch. **b**, Height/thickness of the perovskite + PVK films shown here was (40±2) nm. Thickness of PVK was (10±2) nm without perovskite layer measured in a separate PVK only sample. The perovskite film is smooth with average roughness of 0.8 nm and root-mean-square (RMS) roughness of 1.4 nm

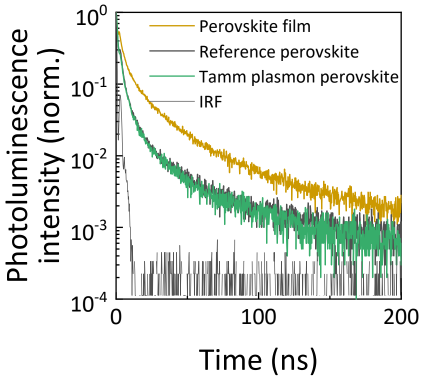

Supplementary Figure 5 | Transient photoluminescence of perovskite structure. Time-resolved photoluminescence of reference-perovskite structure without silver, reference-perovskite structure with silver and Tamm-plasmon-perovskite structure with effective lifetimes of roughly 3.7 ns, 1.5 ns and 1.4 ns respectively. All perovskite films shown in this figure are quasi-2D perovskite without 18-crown-6 additive. Samples excited at 5 nJ cm^{-2} $pulse^{-1}$ with Edinburgh Instruments FLS1000. Effective lifetime is defined as the time required for photoluminescence intensity reduces to e^{-1} of the initial intensity.

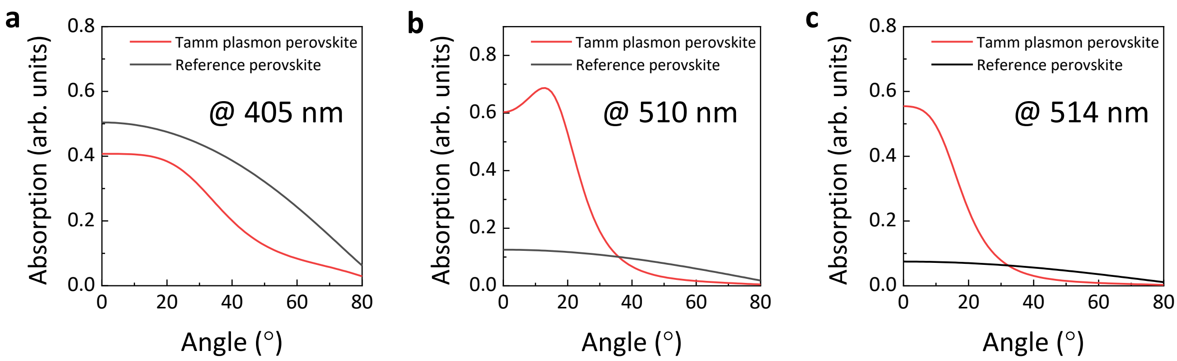

Supplementary Figure 6 | Simulated absorption profile of perovskite in Tamm-plasmon-perovskite structure. The absorption profile of perovskite layer at **(a)** 405 nm (excitation wavelength), **(b)** 510nm (quasi-2D perovskite emission wavelength) and **(c)** 514 nm in reference-perovskite and narrow-angle-Tamm-plasmon-perovskite structure.

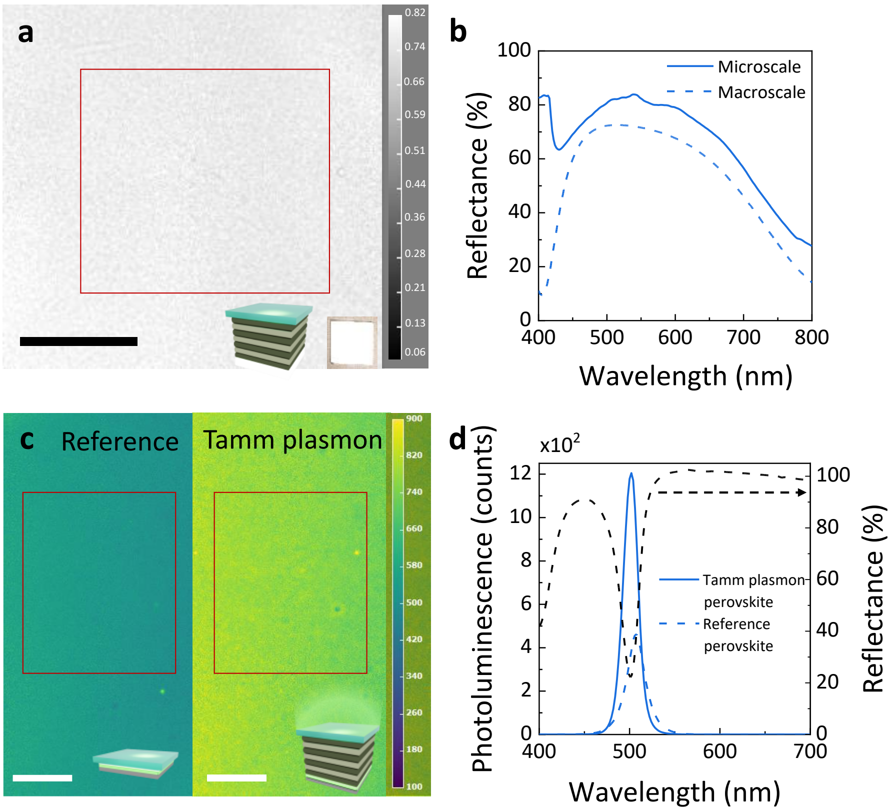

Supplementary Figure 7 | Microscopic imaging. a, Homogenous reflectance across photonic crystals substrate measured under the hyperspectral microscope. The scale bar shows 50 µm. Inset: picture of smooth and shiny photonic crystals substrate and schematic of photonic crystals substrate. **b,** Average reflectance over the boxed area compared with macroscale reflectance. **c,** Microscopic photoluminescence of reference and narrow-angle-Tamm-plasmon-perovskite structure (schematics shown as inset) measured under the hyperspectral microscope with collection angle of 26.7° (objective lens numerical aperture, NA = 0.45, more details in Method). Both scale bars show 50 µm. **d,** Average photoluminescence of reference and Tamm-plasmon-perovskite structure across boxed region. Tamm plasmon resonance wavelength shown in black dashed line.

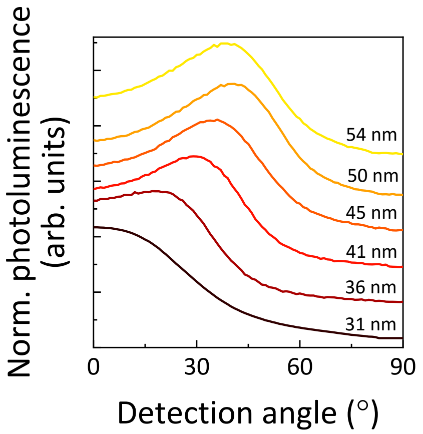

Supplementary Figure 8 | Tuneable directionality for wide-angle-Tamm-plasmon-perovskite structure. Normalized integrated photoluminescence across detection angle of wide-angle-Tamm-plasmon-perovskite samples with perovskite thickness increasing from 31 nm, 36 nm, 41 nm, 45 nm, 50 nm, and 54 nm.

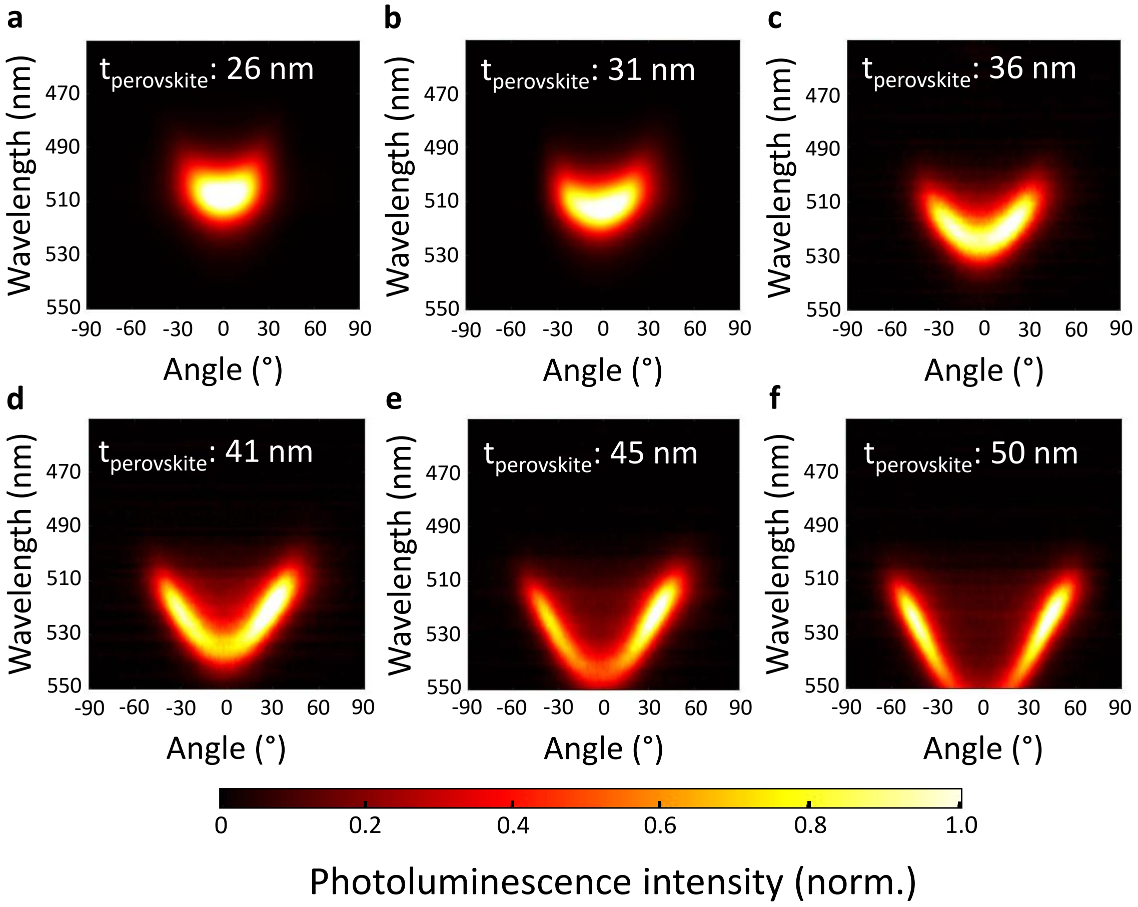

Supplementary Figure 9 | Angular dependent photoluminescence spectra of Tamm-plasmon-perovskite structure. Normalized photoluminescence spectra collected across the Tamm-plasmon-perovskite sample surface with quasi-2D perovskite thickness of **(a)** 26 nm (narrow-angle-Tamm-plasmon shown in Figure 2d-f), **(b)** 31 nm, **(c)** 36 nm, **(d)** 41 nm, **(e)** 45 nm and **(f)** 50 nm (wide-angle-Tamm-plasmon shown in Figure 2g-i).

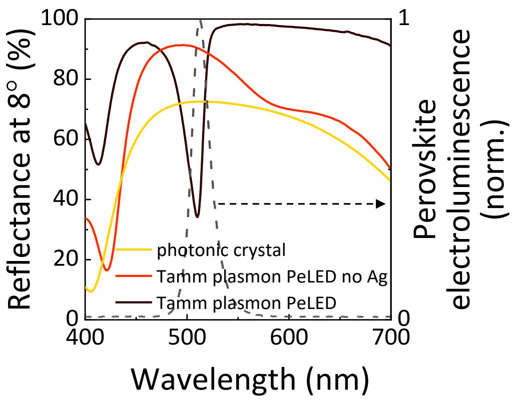

Supplementary Figure 10 | Experimental optimisation of narrow-angle-Tamm-plasmon PeLED. Measured 8° reflectance of photonic crystal, Tamm plasmon PeLED no silver (ag) (glass/photonic crystal/poly(N,N'-bis-4-butylphenyl-N,N'-bisphenyl)benzidine(poly-TPD)/PVK/quasi-2D perovskite/2,2',2"-(1,3,5-Benzinetriyl)-tris(1-phenyl-1-H-benzimidazole)(TPBi)/(8-Hydroxyquinolinato)lithium (LiQ) and full narrow-angle-Tamm plasmon PeLED structure (glass/photonic crystal/poly-TPD/PVK/quasi-2D perovskite/TPBi/LiQ/Ag. The Tamm plasmon PeLED no Ag structure shows a photonic stopband with up to 90% reflectance at the centre wavelength which is near the perovskite electroluminescence and the narrow-angle-Tamm-plasmon PeLED resonance wavelength.

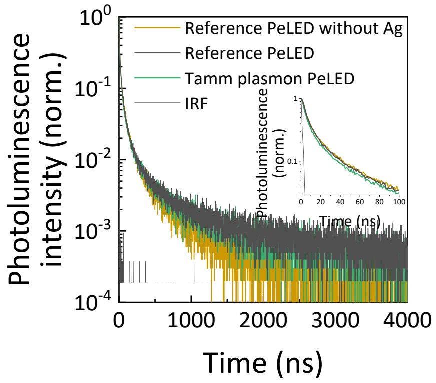

Supplementary Figure 11 | Transient photoluminescence of PeLED structure. Time-resolved PL of reference PeLED without silver (Ag), reference PeLED and Tamm-plasmon-driven PeLED with effective lifetimes of roughly 10 ns, 10 ns and 8.5 ns respectively. All perovskite films shown in this figure are quasi-2D perovskite with 18-crown-6 additive. Samples excited at 5 nJ cm^{-2} pulse^{-1} with Edinburgh Instruments FLS1000. Effective lifetime is defined as the time required for PL intensity reduces to e^{-1} of the initial intensity.

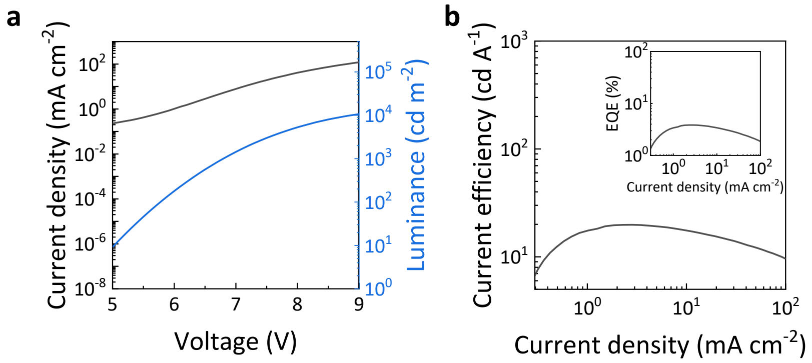

Supplementary Figure 12 | Wide-angle-Tamm-plasmon-driven PeLED performance. a, Current density-Voltage-Luminance (blue line). **b,** Current efficiency and EQE (inset) vs current density.

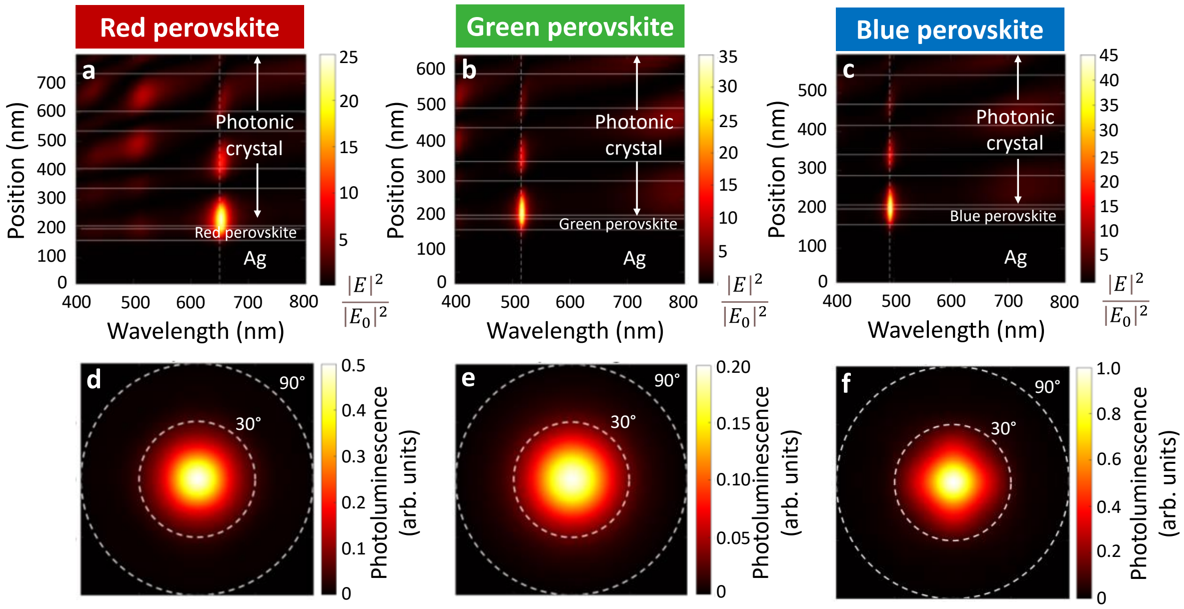

Supplementary Figure 13 | Tamm-plasmon-perovskite structure for red, green and blue regime. The structure of **(a,d)** red, **(b,e)** green, and **(c,f)** sky-blue Tamm-plasmon-perovskite structure each optimized with transfer matrix model powered by genetic algorithm. The materials of photonic crystal stack and plasmonic metal, and number of photonic crystal layer pairs are kept constant. Refractive indices of red, green and sky-blue perovskites are shown in Supplementary Fig. 16. **(a-c)** Simulated electric field enhancement of Tamm-plasmon-perovskite structures. **(d-f)** Finite-difference time-domain simulation of angular output power normalized to the PL intensity of sky-blue Tamm-plasmon-perovskite structure (see colour bar).

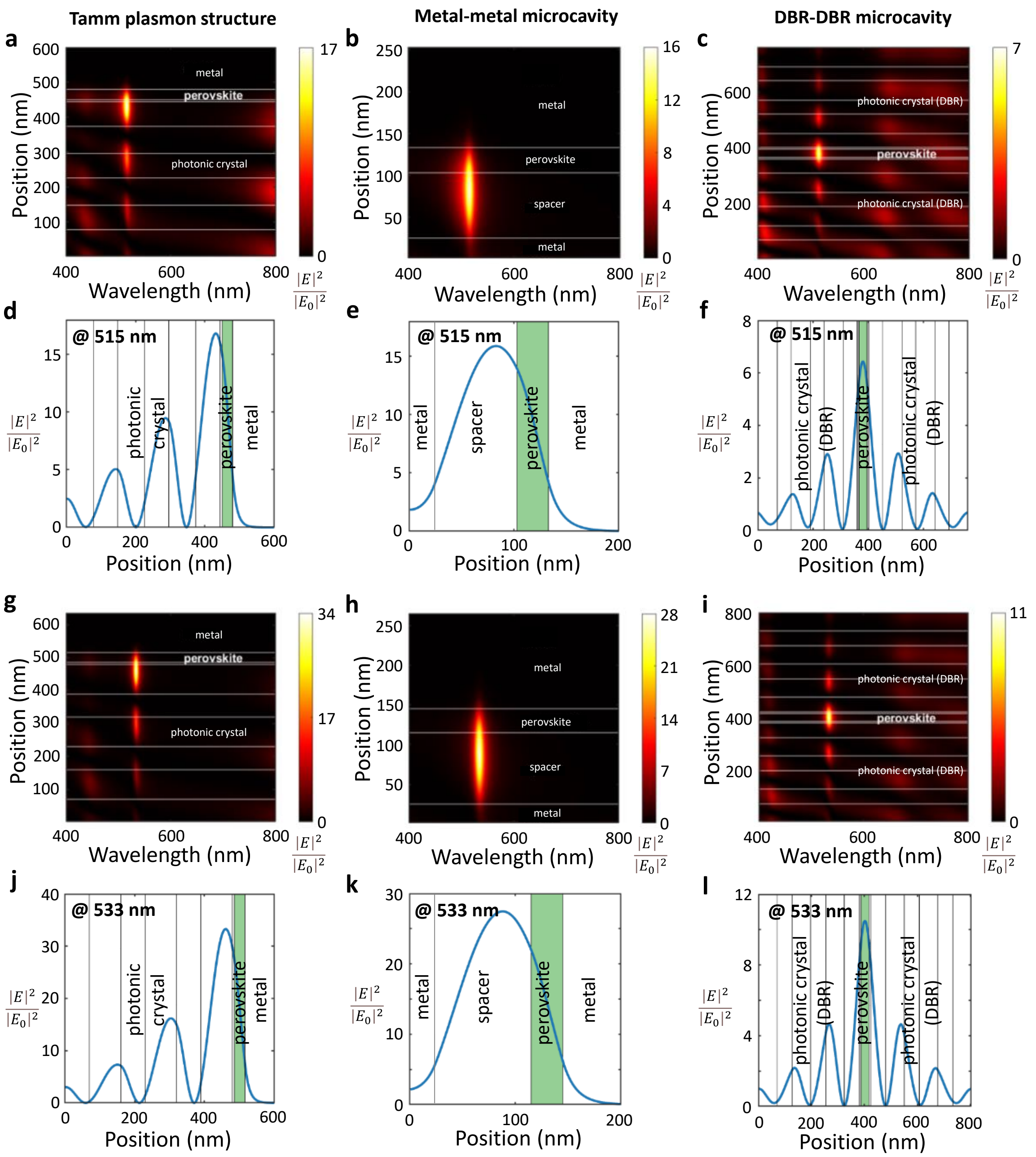

Supplementary Figure 14 | Simulation comparison between Tamm plasmon structure, metal-metal microcavity and distributed Bragg reflector (DBR)-DBR microcavity. Designed structures to support the electric field intensity enhancement for **(a-f)** λ = 515 nm and **(g-l)** λ = 533 nm: Simulation of electric field intensity enhancement across the cross-section **(a-c, g-i)** and electric field enhancement as a function of the position within the structure **(d-f, j-l)** of a Tamm plasmon perovskite (30 nm) structure **(a,d,g,j)**, a perovskite (30 nm) in metal (25 nm) - metal (120 nm) microcavity with spacer, **(b,e,h,k)** and a perovskite (30 nm) in DBR-DBR microcavity **(c,f,i,l)**. The green stripe shows the perovskite layer. Each of the three systems has conditions set to align the maximum electric field intensity within the perovskite layer.

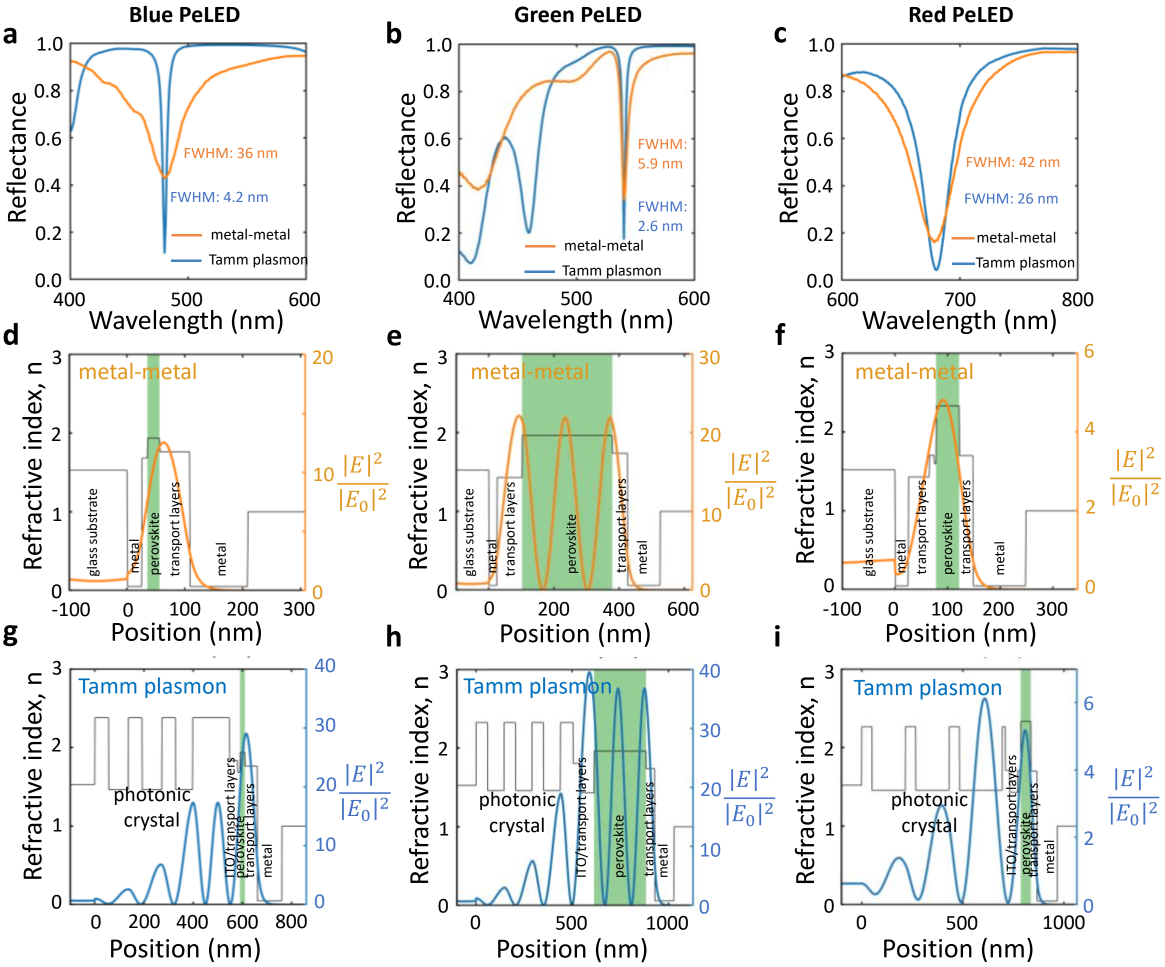

Supplementary Figure 15 | Simulated Tamm plasmon and metal-metal cavities incorporating high-efficiency device structures in **(a,d,g)** blue [2], **(b,e,h)** green [3] and **(c,f,i)** red [4] perovskite LEDs. **(a-c)** Resonance of Tamm plasmon structures and metal-metal cavities. The Tamm plasmon structure is simulated following the device structures reported. The thicknesses of the device structure of metal-metal cavities are multiplied by a constant factor (blue: 0.88; green: 1.01; red: 0.85) to ensure the resonance matches the reported electroluminescence. By minimising changes to the device structure thickness ratio, we ensure minimum changes to electrical device performance (e.g. charge balance from the reported papers). **(d-f)** Refractive index **(black line)** and electric field intensity **(orange line)** across the metal-metal cavity structures. **(g-i)** Refractive index **(black line)** and electric field intensity **(blue line)** across the Tamm plasmon structures. The green stripe shows the perovskite layer. To realise good electric field confinement in the Tamm plasmon structure, the number of photonic crystal layer pairs is tuned and an extra TiO_2 layer is added as shown in **(g-i)** to match the optical properties of the hole-transport layer/perovskite/electron-transport layer in the reported device structure.

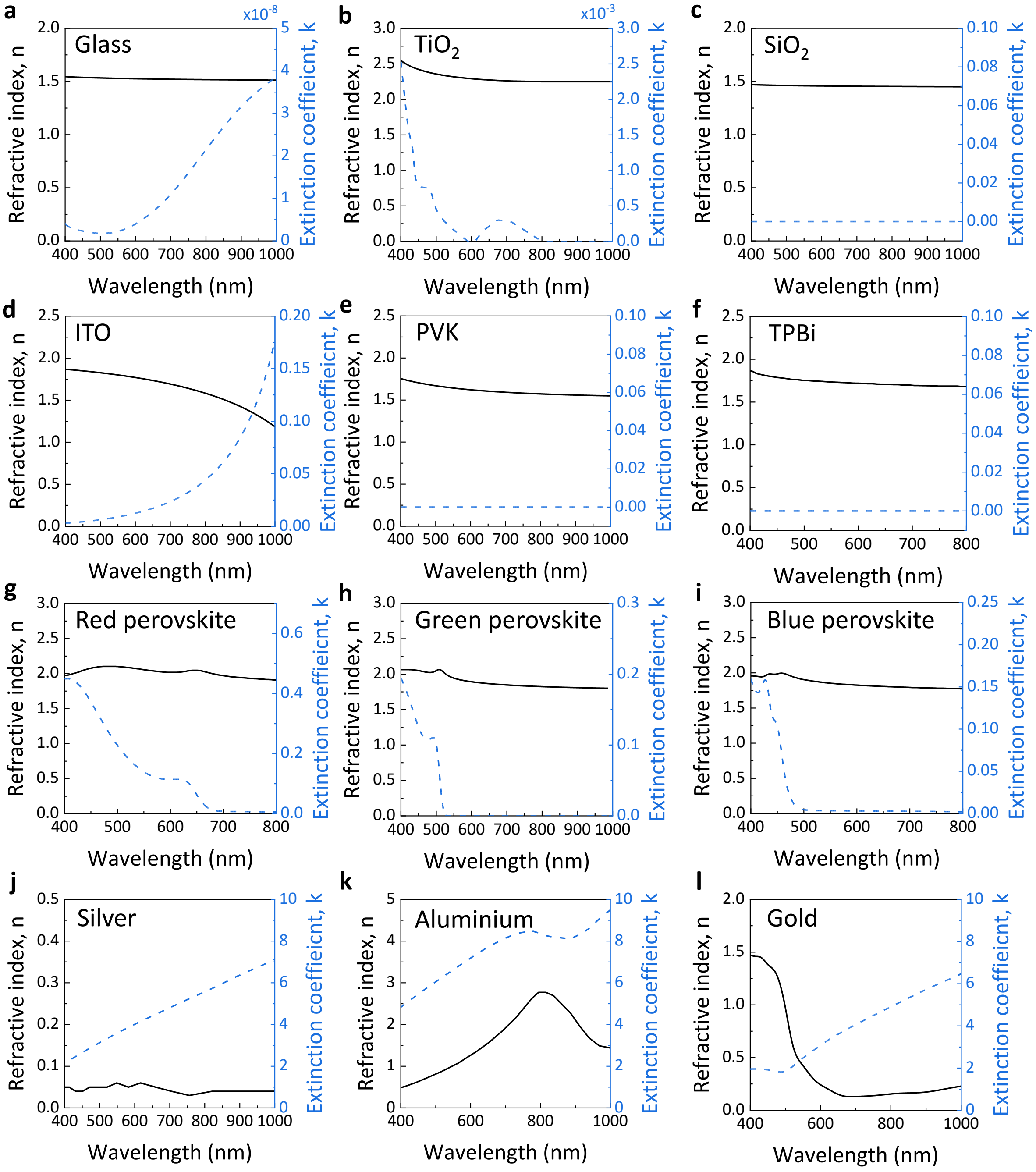

Supplementary Figure 16 | Optical constant of materials used in simulations. Real refractive index, *n* and extinction coefficient, *k* of **(a)** Glass *, **(b)** TiO_2 *, **(c)** SiO_2 *, **(d)** ITO [5], **(e)** PVK [6], **(f)** TPBi [5], **(g)** red perovskite [7], **(h)** green perovskite *, **(i)** sky blue perovskite [7], **(j)** Ag [8], **(k)** Al [9] and **(l)** Au [8]. * measured by ellipsometry

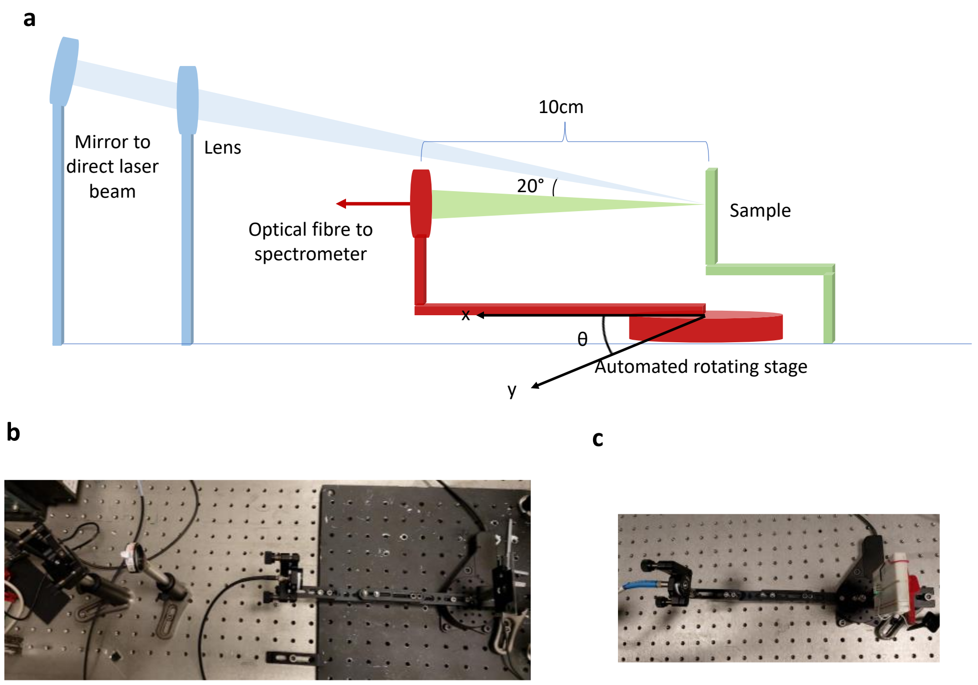

Supplementary Figure 17 | Angular photoluminescence and electroluminescence setup. a, Shown in blue are optics to focus 405 nm excitation laser on sample. Sample was excited at roughly 20° above the sample and normal to the sample at **x-y** plane. The sample holder (in green) was fixed on the optical table with sample placed directly above the centre of the rotating stage. The fibre collimator connecting to the detector (in red) was aligned perpendicular to the sample in **z**-plane and rotated 180° around the sample at 100 mm radius. **b,** Picture of mirror, lens, fibre collimator, rotation stage and sample holder of angular PL setup. **c,** Picture of fibre collimator, rotation stage and sample holder of angular EL setup.

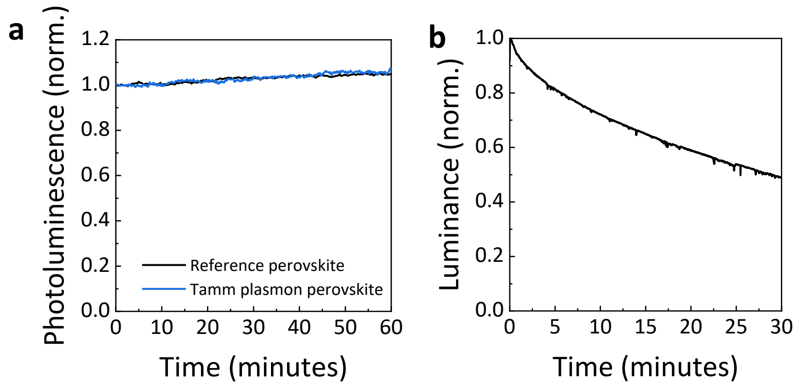

Supplementary Figure 18 | Stability test. a, Normalized photoluminescence integrated across the spectra of reference perovskite structure and Tamm plasmon perovskite structure, measured across an hour excited at 405 nm with power density of 0.5 W cm^{-2} (same power density as angular photoluminescence measurement). **b,** Normalized luminance of PeLED measured at constant current density of 0.44 mA cm^{-2} (same current density as angular electroluminescence measurement) showing T_{90} of 100 seconds, which is sufficient for a complete angular electroluminescence measurement which takes 36 seconds for a 180° rotation cycle. The LED is driven at relatively high current to produce high luminance for sufficient signal-to-noise ratio collected by the detector that is placed 10 cm away from the sample.

Supplementary Note 1 | Photonic crystal with stopband around the visible region

In a Tamm plasmon system, the photonic crystal layer at the metal-photonic-crystal interface must be the layer with higher refractive index [10]. In our design, we employ alternating TiO_2/SiO_2 layers as the photonic crystal because they are uniform, stable and robust with optical stopband within the visible range [11]. The refractive index of TiO_2 and SiO_2 are n = 2.35 and n = 1.46 at 510 nm respectively (Supplementary Fig. 16), thus TiO_2 is initially the higher refractive index layer at the metal-photonic-crystal interface. The refractive index of TiO_2 is comparable to the refractive index of the quasi-2D perovskite (n = 2.05 at 510 nm) (Supplementary Fig. 16). This allows a good replacement of the TiO_2 layer at the metal-photonic crystal-interface with a quasi-2D perovskite layer to retain the photonic crystal bandgap while introducing an emitter layer to the Tamm plasmon system. With customized thickness of 1-dimensional photonic crystal composed of alternating TiO_2 and SiO_2 layers, we achieve a photonic stopband between 450 nm to 630 nm, which is comparable to our simulated optimized design (Supplementary Fig. 1). The spectral properties of the photonic crystal are very homogenous with uniform optical stopband observed both macro- and microscopically (see Supplementary Fig. 7). As Tamm plasmons are formed at the metal-photonic-crystal interface, with a good replacement of TiO_2 with quasi-2D perovskite layer as the high refractive index layer at the metal-photonic crystal interface, we show in Supplementary Fig. 1 that the photonic crystal + perovskite structure forms distributed Bragg reflector (DBR) with up to >85% reflectance with centre wavelength at the desired Tamm plasmon resonance wavelength (the perovskite photoluminescence wavelength). Similar design procedures are implemented for the Tamm plasmon PeLEDs by optimising the photonic crystal + perovskite + device layers to achieve a good DBR. In Supplementary Fig. 10, we showed the reflectance of Tamm plasmon PeLED without Ag structure with photonic stopband of reflectance up to 90% centred at the desired Tamm plasmon resonance (the perovskite electroluminescence wavelength).

In the Tamm plasmon system, it is favourable to place the emitting layer as close to the metal-photonic-crystal interface as possible for highest confinement of electric fields. However, in the microcavities, as standing waves form between the two mirrors, careful design is required to position the emitting layer at the antinode by adding spacer/fillers or good control of transport layer thickness to maximise the confinement of fields.

Supplementary Note 2 | Photoluminescence quantum efficiency (PLQE)

In Fig. 2, the reference and Tamm plasmon perovskite structure has quasi-2D perovskite without 18-crown-6 as additive as the emitting material. The PLQE of the quasi-2D perovskite film without 18-crown-6 additive is 5±1% at excitation fluence of 33 mW cm^{-2}. However, due to strong metal quenching, the PLQE of reference perovskite (glass/PVK/quasi-2D perovskite/Ag) and Tamm plasmon perovskite structure (glass/photonic crystal/PVK/quasi-2D perovskite/Ag) are both <1% at excitation fluence of 33 mW cm^{-2}.

In Fig. 3, the reference and Tamm plasmon PeLEDs have quasi-2D perovskite with 18-crown-6 additive as the emitting layer, where the 18-crown-6 is added to improve the LED performance. The PLQE of the quasi-2D perovskite film with 18-crown-6 additive is 47±2%, while the PLQE of reference PeLED stack and Tamm plasmon PeLED stack is 6±1% and 3±1% respectively all at excitation fluence of 33 mW cm^{-2}. The PLQE of reference PeLED and Tamm plasmon PeLED stack drops compared to the quasi-2D perovskite film with 18-crown-6 due to transport layer and metal quenching.

Supplementary Note 3 | Roll-on and roll-off quasi-2D PeLED efficiency

Similar to our observations in both reference PeLED and Tamm plasmon PeLED shown in Fig. 3c and Supplementary Fig. 12, quasi-2D PeLED has shown stronger roll-on and roll-off in efficiency compared to their 3D bulk counterparts. The stronger roll-on is mainly due to efficient funnelling and confinement of charge carriers in naturally formed quantum well-like structures with a mixed number of inorganic octahedral layers (see Supplementary Fig. 2); while the strong roll-off at high current density is likely due to charge injection imbalance, Auger-induced luminescence quenching and migration of organic ligands [12–15]. This is still a matter of investigation in the field, which is of our interest but not within the specific scope of this work.

Supplementary Note 4 | Transient photoluminescence change in reference PeLED with and without silver

In Supplementary Fig. 11, we compare the transient photoluminescence of reference PeLED with and without Ag (black and yellow line, respectively) and a Tamm plasmon PeLED (green line). Firstly, as all structures include an electron transport layer (TPBi/LiQ), which acts as a spacer between the perovskite film and Ag, we observe very similar PL lifetime in all samples as the metal quenching becomes less dominant than in the cases shown in Supplementary Fig. 5 [16]. Moreover, the photoluminescence lifetime tail becomes slightly longer for the PeLEDs with Ag, possible due to a better reflection of light which could vary the photon density within the perovskite film, thus affecting the lifetime [17].

Supplementary References

1. Yuan, M. *et al.* Perovskite energy funnels for efficient light-emitting diodes. *Nat. Nanotechnol.* **11**, 872–877 (2016).
2. Yuan, S. *et al.* Efficient blue electroluminescence from reduced-dimensional perovskites. *Nat. Photonics* 1–7 (2024) doi:10.1038/s41566-024-01382-6.
3. Kim, J. S. *et al.* Ultra-bright, efficient and stable perovskite light-emitting diodes. *Nature* **611,** 688–694 (2022).
4. Jiang, J. *et al.* Red Perovskite Light-Emitting Diodes with Efficiency Exceeding 25% Realized by Co-Spacer Cations. *Adv. Mater.* **34**, 2204460 (2022).
5. Shin, M. *et al.* Understanding the Origin of Ultrasharp Sub-bandgap Luminescence from Zero-Dimensional Inorganic Perovskite Cs4PbBr6. *ACS Appl. Energy Mater.* **3**, 192–199 (2020).
6. Bermudez, V. *et al.* Linear and nonlinear optical properties of polyvinyl carbazole and polyvinyl-carbazole-substituted thin films. in *Linear, Nonlinear, and Power-Limiting Organics* vol. 4106 165–176 (SPIE, 2000).
7. Chen, Z. *et al.* Utilization of Trapped Optical Modes for White Perovskite Light-Emitting Diodes with Efficiency over 12%. *Joule* **5**, 456–466 (2021).
8. Johnson, P. B. & Christy, R. W. Optical Constants of the Noble Metals. *Phys. Rev. B* **6**, 4370–4379 (1972).
9. Rakić, A. D. Algorithm for the determination of intrinsic optical constants of metal films: application to aluminum. *Appl. Opt.* **34**, 4755–4767 (1995).
10. Kaliteevski, M. *et al.* Tamm plasmon-polaritons: Possible electromagnetic states at the interface of a metal and a dielectric Bragg mirror. *Phys. Rev. B* **76**, 165415 (2007).
11. Persano, L. *et al.* Very high-quality distributed Bragg reflectors for organic lasing applications by reactive electron-beam deposition. *Opt. Express* **14**, 1951 (2006).
12. Ban, M. *et al.* Solution-processed perovskite light emitting diodes with efficiency exceeding 15% through additive-controlled nanostructure tailoring. *Nat. Commun.* **9**, 3892 (2018).
13. Zou, C., Liu, Y., Ginger, D. S. & Lin, L. Y. Suppressing Efficiency Roll-Off at High Current Densities for Ultra-Bright Green Perovskite Light-Emitting Diodes. *ACS Nano* **14**, 6076–6086 (2020).
14. Warby, J. H. *et al.* Revealing Factors Influencing the Operational Stability of Perovskite Light-Emitting Diodes. *ACS Nano* **14**, 8855–8865 (2020).
15. Cheng, T. *et al.* Ion Migration-Induced Degradation and Efficiency Roll-off in Quasi-2D Perovskite Light-Emitting Diodes. *ACS Appl. Mater. Interfaces* **12**, 33004–33013 (2020).
16. Amos, R. M. & Barnes, W. L. Modification of the spontaneous emission rate of Eu3+ ions close to a thin metal mirror. *Phys. Rev. B* **55**, 7249–7254 (1997).
17. Raja, W. *et al.* Photon recycling in perovskite solar cells and its impact on device design. *Nanophotonics* **10**, 2023–2042 (2020).
